# Orbital migration of schistosome eggs: a case report

**DOI:** 10.1186/s12886-021-01956-w

**Published:** 2021-04-27

**Authors:** Nouhoum Guirou, Serge Resnikoff, Abba Kaka Hadja Yakoura, Michel Gouda, Seydou Bakayoko, Abdoulaye Napo, Rodrigue Romulad Elien, Robert Della Rocca, Fatoumata Sylla, Lamine Traoré

**Affiliations:** 1 CHU-IOTA/ Université des sciences des techniques et des technologies de Bamako, Bamako, Mali; 2 Brien Holden Vision Institute, University of New South Wales, Sydney, Australia; 3 Hôpital National de Niamey, Niamey, Niger; 4 New York Eye and Ear Infirmary of Mount Sinai, New York, USA

**Keywords:** Orbit, Schistosoma, Neglected tropical disease, Lacrimal gland, Case report

## Abstract

**Background:**

Ocular damage, including damage to the conjunctiva, lacrimal gland, eyelids, and orbit, caused by *Schistosoma haematobium* is sporadic. We report a clinical case of orbital migration of schistosome eggs.

**Case presentation:**

A 14-year-old boy of Malian nationality presented with a painless swelling of the upper right eyelid, which had been gradually increasing for approximately 3 months. Visual acuity was logMAR 0.10 and 0.00 in the right and left eye, respectively. External examination revealed a right palpebral mass, pushing the globe slightly downward and inward. Computed tomography revealed a mass of the right lacrimal gland. Total excision of the mass was performed by transpalpebral orbitotomy. Pathological examination revealed an inflammatory granulomatous infiltrate of the lacrimal gland consisting of lymphocytes, eosinophils, giant cells, epithelioid cell, histiocytes and calcified Schistosoma eggs with terminal spine.

Urine examination revealed eggs of *S. haematobium*. Praziquantel 40 mg/kg was administered to the patient. The hematuria stopped after 1 week. After 3 years of follow-up, no recurrence was noted.

**Conclusions:**

The bilharzian granuloma of the lacrimal gland is an ectopic site of the parasite. In this case, the granuloma was cured by surgical excision followed by a course of Praziquantel.

## Background

Neglected tropical diseases (NTDs) are a diverse group of bacterial, viral, parasitic, fungal, and non-communicable origin diseases [1]. These diseases affect mainly tropical areas, in the neglected and resource-poor communities. Currently, the control efforts for these diseases focus on approximately 20 NTDs, including schistosomiasis [1, 2].

*Schistosoma mansoni* (*S. mansoni*) and *Schistosoma haematobium* (*S. haematobium)* are the two most common types of schistosomes in Africa. The preferred site for *S. mansoni* and *S. haematobium* is the digestive tract and urogenital tract, respectively. Ectopic forms of schistosomiasis, most of which are secondary to *S. mansoni* and *Schistosoma japonicum,* are found in the digestive, urogenital, central nervous, cardiovascular, pulmonary, dermatological, and ophthalmological systems [3–6].

Ocular damage, including damage to the conjunctiva, lacrimal gland, eyelids, and orbit, caused by *S. haematobium* is sporadic [7–14]. Ocular manifestations are dominated by inflammation and granuloma [8, 10]. Granulomatous involvement of the lacrimal gland was first described in 1977. Herein, we report a clinical case of orbital migration of schistosome eggs in a 14-year-old boy.

## Case presentation

**Fig. 1:**
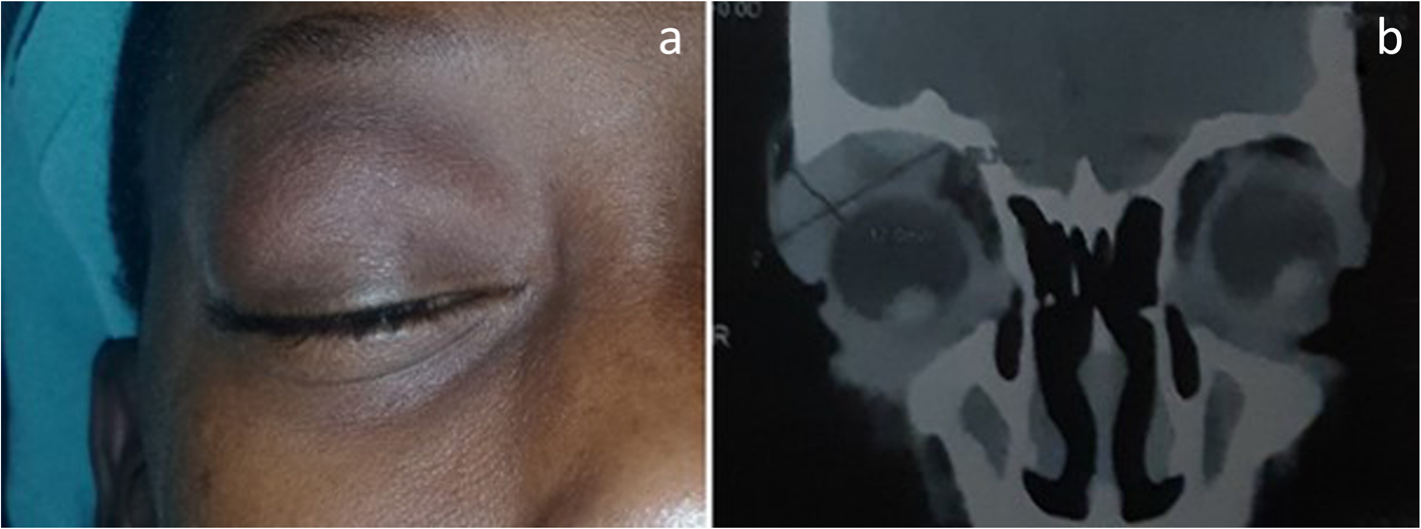
**a** orbito-palpebral mass, pushing the eyeball slightly downward and inward, with grade 1 exophthalmos (patient under GA). **b** Computed tomography: Right lacrimal gland mass measuring 29 × 13 mm with low tissue density and heterogeneity that was enhanced by iodine contrast

A 14-year-old boy of Malian nationality accompanied by his parents presented at IOTA, Bamako, Mali, with a painless swelling of the upper right eyelid, which had been gradually increasing for approximately 3 months. Visual acuity was logMAR 0.10 and 0.00 in the right and left eye, respectively. External examination revealed a right orbito-palpebral mass, pushing the globe slightly downward and inward (Fig. 1a). Slit lamp examination of the anterior segment and fundus was normal in both the eyes; intraocular pressure was 13 mmHg on both eyes. Computed tomography revealed a mass of the right lacrimal gland with low tissue density and heterogeneity that was enhanced by iodine contrast. The right palpebral mass measured 29 × 13 mm (Fig. 1b).

**Fig. 2:**
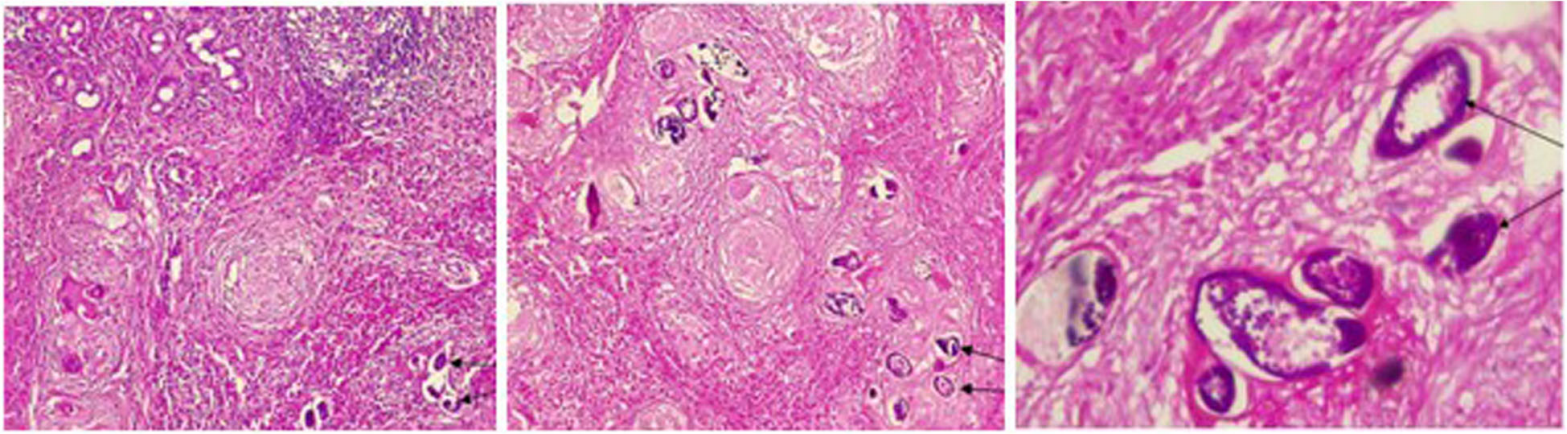
Lacrimal gland granuloma with lymphocytes, eosinophil, giant cells, epithelioid cell, histiocytes and calcified schistosome eggs (arrows) with terminal spine (magnification 100–200-300 X)

Total excision of the mass was performed by transpalpebral orbitotomy. Pathological examination revealed an inflammatory granulomatous infiltrate of the lacrimal gland consisting of lymphocytes, eosinophils, giant cells, epithelioid cell, histiocytes and calcified schistosome eggs with terminal spine (Fig. 2).

**Fig. 3:**
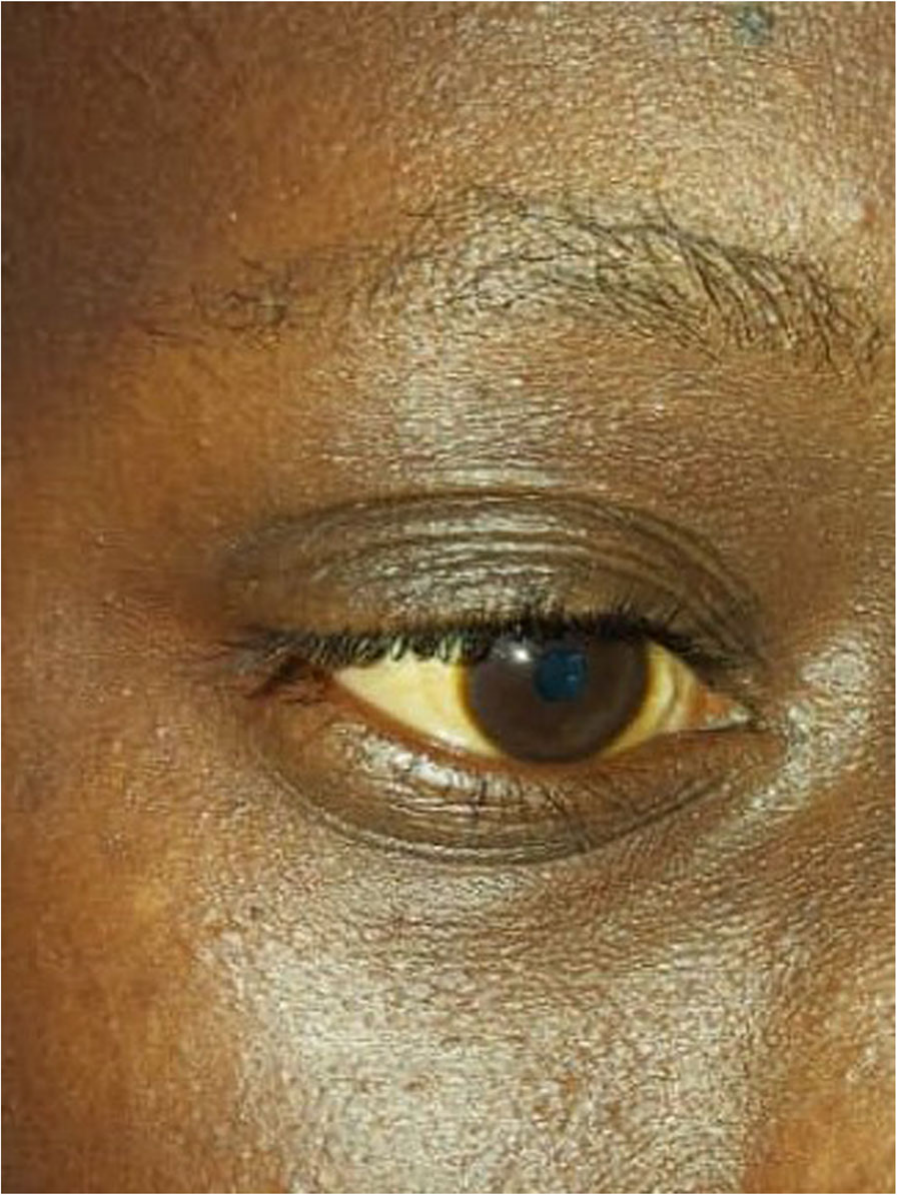
No recurrence after 3 years follows up

We questioned the patient and his parents regarding a possible history of schistosomiasis. The patient revealed that he has been experiencing terminal hematuria for at least 3 months. Urine examination revealed eggs of *S. haematobium*. An abdominopelvic ultrasound found irregular thickening of the bladder wall with cystitis and homogeneous splenomegaly. No other ectopic locations of the eggs were found. Praziquantel 40 mg/kg was administered to the patient. The hematuria stopped after 1 week. The dose of the prescribed medication was renewed 1 month later. After 3 years of follow-up, no recurrence was noted (Fig. 3).

## Discussion and conclusions

In Mali, the estimated prevalence of *S. haematobium* is 38.4% among school-aged children [15]. The areas of endemic bilharzian diseases are mainly along the Niger River. Our case was residing in this area (Macina) at the time of diagnosis.

Adult male and female worms of *S. haematobium* are found in the vascular system in humans [6]. They lay eggs that are then released into the vesical venous plexus before being expelled in the urine.

Ophthalmic damage caused by *S. haematobium* was first described in 1927 in the conjunctiva [13]. Other localizations, particularly in the eyelids, orbit, and optic nerve, have also been described [7, 10, 12, 13]. Granulomatous involvement of the lacrimal gland was first described in 1977 by Jakobiec [10]. The patient reported was a 11-year-old boy from Sierra Leone. The pathological description of our patient’s surgical specimen was similar to that as described by Jakobiec.

In the case reported by Jakobiec, adult worms were found simultaneously along with the eggs [9, 16]. In our case, no worm was found.

Only 20 to 55% of the eggs are successfully excreted, while the rest are inevitably trapped in the host tissues. Schistosome eggs eventually pass through the mesenteric vessels [6, 16, 17].

Consequently, the infiltrating eggs are focal points for the host immune system, which sets up a distinct attack and sequestration strategy in response: the granuloma. The formation of granulomas around the eggs is a consequence of a delayed hypersensitivity reaction [18].

Granulomas are highly organized multicellular structures that are enriched with a range of immune cells, including T helper type 2 cells, macrophages, and eosinophils, with mast cell infiltration and accumulation of type 2 cytokines [16, 18].

The newly laid eggs are immediately covered with cells and proteins from the hemostatic system. These haemostatic constituents promote their anchoring to the endothelium and prevent them from being transported by the bloodstream [16].

The granuloma is both beneficial and harmful to the host. On one hand, granulomas protect host tissues from toxins released by the eggs. On the other hand, fibrous sequelae following granuloma resolution are the major cause of morbidity and mortality in schistosomiasis [16].

The World Health Organization recommends the use of praziquantel for the treatment of *S. haematobium* [17]. In our case, two subsequent doses of praziquantel resulted in a complete cure.

Orbital migration of *S. haematobium* eggs is infrequent, especially to the lacrimal gland. The bilharzian granuloma of the lacrimal gland is an ectopic site of the parasite. In this case, the granuloma was cured by surgical excision followed by a course of Praziquantel.

## Data Availability

All data generated or analyzed during this study are included in this published article.

## Abbreviations

NTDs: Neglected tropical diseases

## References

[1] Hotez PJ, Kamath A (2009). Neglected tropical diseases in sub-Saharan Africa: review of their prevalence, distribution, and disease burden. PLoS Negl Trop Dis.

[2] Jannin J, Solano P, Quick I, Debre P (2017). The francophone network on neglected tropical diseases. PLoS Negl Trop Dis.

[3] Imai K, Koibuchi T, Kumagai T, Maeda T, Osada Y, Ohta N (2011). Cerebral schistosomiasis due to Schistosoma haematobium confirmed by PCR analysis of brain specimen▿. J Clin Microbiol.

[4] Chassot CA, Christiano CG, Barros MS, Rodrigues CJ, Corbett CEP (1998). Colon polyps in Schistosoma haematobium schistosomiasis. Mem Inst Oswaldo Cruz.

[5] Poderoso WLS, de Santana WB, da Costa EF, Cipolotti R, Fakhouri R (2008). Ectopic schistosomiasis: description of five cases involving skin, one ovarian case and one adrenal case. Rev Soc Bras Med Trop.

[6] Colley DG, Bustinduy AL, Secor WE, King CH (2014). Human schistosomiasis. Lancet.

[7] Loridant S, Mouahid G, Cornu M, Allienne J-F, Leroy J, Maurage C-A (2018). Case report: hemianopia: from suspected glioblastoma to the diagnosis of ectopic schistosomiasis Haematobium infection in a traveler returning from the Republic of the Congo. Am J Trop Med Hyg.

[8] Milligan A, Burns DA (1988). Ectopic cutaneous schistosomiasis and schistosomal ocular inflammatory disease. Br J Dermatol.

[9] Abboud IA, Hanna LS, Ragab HA (1971). Experimental ocular schistosomiasis. Br J Ophthalmol.

[10] Jakobiec FA, Gess L, Zimmerman LE (1977). Granulomatous dacryoadenitis caused by Schistosoma haematobium. Arch Ophthalmol.

[11] Ismail HIH, Ashour DS, Abou Rayia DM, Ali AL (2016). Ocular pathological changes in hamsters experimentally infected with *Schistosoma mansoni*. J Helminthol.

[12] Gilles M, Combillet F, Alberti N, Malvy D, Korobelnik J-F (2016). Orbitopathie inflammatoire révélatrice d’une bilharziose systémique. J Fr Ophtalmol.

[13] Badir G (1946). Schistosomiasis of the conjunctiva. Br J Ophthalmol.

[14] Randriamora JTM, Rabarijaona HZ, Rabearivony N, Bernardin P, Rasoavelonoro VA (2004). Tumeur sous-conjonctivale bulbaire révélant un granulome bilharzien périoculaire. J Fr Ophtalmol.

[15] Clements ACA, Bosqué-Oliva E, Sacko M, Landouré A, Dembélé R, Traoré M (2009). A comparative study of the spatial distribution of schistosomiasis in Mali in 1984–1989 and 2004–2006. PLoS Negl Trop Dis.

[16] Costain AH, MacDonald AS, Smits HH (2018). Schistosome Egg Migration: Mechanisms, Pathogenesis and Host Immune Responses. Front Immunol.

[17] Nelwan ML (2019). Schistosomiasis: life cycle, diagnosis, and control. Curr Ther Res Clin Exp.

[18] Domingo EO, Warren KS (1970). Granuloma Formation around *Schistosoma mansoni*, S. Haematobium, and *S. japonicum* Eggs: Size and rate of development, cellular composition, cross-sensitivity, and rate of egg destruction. Am J Trop Med Hyg.

